# Bridging science and art: Auralization and visualization of the ocean soundscape

**DOI:** 10.1016/j.isci.2024.110696

**Published:** 2024-08-30

**Authors:** Yu Ren, Tian Wu, Chuanzhi Li, Pengfei Yao, Weiwei Ding

**Affiliations:** 1 GEOMAR Helmholtz Centre for Ocean Research Kiel, Kiel, Germany; 2 Muthesius University of Fine Arts and Design, Kiel, Germany; 3 Ocean University of China, Qingdao, China; 4 Deep Sea Light Team, Kiel, Germany; 5 Key Laboratory of Submarine Geosciences, Second Institute of Oceanography, Ministry of Natural Resources, Hangzhou, China; 6 School of Oceanography, Shanghai Jiao Tong University, Shanghai, China

## Abstract

As a group of researchers and artists, we have collaborated on a science-art project that converts seismic and hydroacoustic data into immersive digital artworks, revealing the hidden realm of ocean soundscape. We integrated artistic methodologies to produce soundtracks and videos from seismic waveforms recorded in the ocean, which enables the general public to gain a multisensory experience of the scientific data studied by specialists. Through exhibitions in multiple venues, our interdisciplinary approach was well received by diverse audiences, showcasing the potential for creative representations of scientific information.

**Figure:**
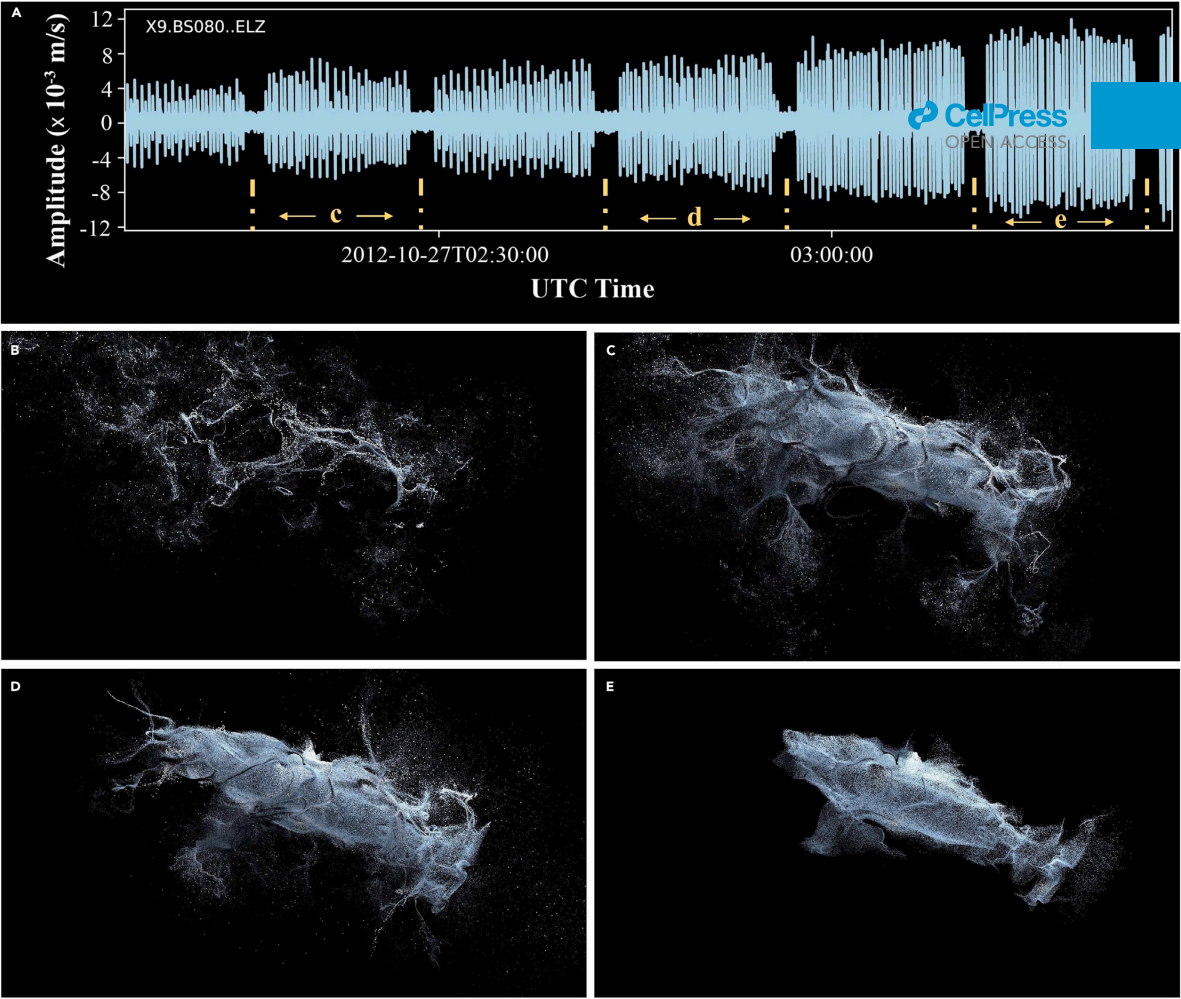
Above image: Digital art images visualizing fin whale songs in the northeast Pacific Ocean. (A) Seismic time series data of the fin whale song recorded at the vertical component (ELZ) of OBS station BS080 under the network code X9. Labels and broken lines indicate short segments of the whale song used to produce art images in c–e. Note that b corresponds to an earlier time window representing weaker energy level and lower amplitude of the seismic waveform prior to the recordings in A. (B–E) Art visuals created based on the variation in the amplitudes of the seismic waveforms. The higher the amplitude, the greater the energy level of the whale song, resulting in a more accurate silhouette of the fin whale.

Our artworks provide new dimensions of scientific data representation, while the creation of the artworks is also driven by the analysis of scientific datasets.

We hope to further integrate earth science and digital art in order to generate additional avenues for public engagement and education, ultimately cultivating a stronger bond between human societies and the ever-changing systems of our planet.

## Main text

### Beginnings

Geoscientists commonly use visual representations such as graphs and tables to explain their research to both their colleagues and individuals without specialized knowledge in the field. Nevertheless, visualizing the mechanisms of our Earth system concealed under intricate geoscientific data has proven to be a difficult task for the geoscience community.^1^ Due to its universal nature, the field of art has experienced significant progress in the utilization of scientific data visualization during the past decades.^2^ The connection between science and art is becoming increasingly important in finding innovative methods to make geoscience more accessible to the public.^3^

A collective of early-career researchers and artists have collaborated on a science-art project known as Deep Sea Light to reveal the hidden realm of sounds in the ocean. This project aims to combine geoscience and digital art in order to create an immersive experience that appeals to a broader audience.^4^ We produced soundtracks and digital art videos depicting various underwater scenarios, such as whale songs, earthquake swarms, and submarine eruptions, detected and extracted from extensive seismic and hydroacoustic datasets. The process of auralization and visualization of the ocean soundscape serves as a connection between the realms of science and art, allowing artists to convert scientific datasets into visually captivating and scientifically stimulating artworks. This interdisciplinary approach not only opens new avenues for scientific investigation and communication but also enhances public engagement. The digital artworks we created were exhibited in several venues in 2023, attracting diverse audiences that included schoolchildren, scholars, artists, politicians, and the general public.

### Art and science

#### The sounds of the ocean

The ocean was assumed to be a quiet place. However, with the increasing number of underwater monitoring experiments,^5^ a hidden realm of sounds in the ocean has been progressively revealed. The soundscape of the ocean is a complex mixture of biotic sounds (e.g., vocalizations from marine mammals and fish), abiotic sounds from natural phenomena like wind and waves, and anthropogenic sounds resulting from human activities. Sounds in the ocean are of utmost importance in the underwater world, exerting a significant influence on the behavior and communication patterns of diverse marine organisms.^6^^,^^7^

Ocean-bottom seismometer (OBS) and hydrophone arrays are commonly used for both seismological research and hydroacoustic monitoring. Between 2011 and 2015, over 300 OBSs were deployed and recovered offshore the Cascadia subduction zone, offering valuable opportunities to study local seismicity,^8^ structures,^9^ and biotic sounds (e.g., blue whale vocalizations^10^) in the northeast Pacific Ocean. While these scientific findings are vital for understanding our Earth system, they can be challenging for individuals without expertise in the field to comprehend and engage with. Taking advantage of the extensive seismic and hydroacoustic datasets, our project aims to explore new dimensions of scientific data representation by integrating artistic methodologies.

#### Creating digital artworks

We first extracted some of the most common sounds recorded by OBS arrays in the northeast Pacific Ocean, for example digital seismic waveform of fin whale calls. These vocalizations consist of frequency-modulated sweeps that decrease in frequency from around 40 to 13 Hz, with a central frequency of approximately 20 Hz. To make the fin whale vocalizations audible to humans, we increased the speed of the digital waveform data (following the study by Kilb et al.^11^) by a factor of 10 (Audio S1).

Audio S1. Fin whale vocalizations recorded at OBS station BS080 in the northeast Pacific Ocean Fin whale calls recorded by seismic stations in the northeast Pacific sped up 10 times to be audible to humans.

Further, we analyzed the spectrums of seismic waveforms and saved them as RGBa visuals. RGBa images consist of pixels, each of which is defined by four parameters: the three R, G, and B values that determine the color, and an alpha channel value that specifies the transparency of that particular pixel. We then utilized the Touch Designer software to convert the RGBa values of the analyzed spectrums into the positions of numerous particles within a three-dimensional xyz coordinate system. Through this approach, we can effectively depict the sounds in a three-dimensional manner and showcase various scenarios to the audience. A series of art images created from seismic waveforms shows the varying levels of discretization in the particles that compose the fin whale’s shape. When the distance between the whale and the OBS station is far away, the particles become dispersed because the energy of the whale calls received by the OBS is relatively low. This corresponds to the relatively low amplitude of the seismic waveforms prior to the beginning time of the seismic whale song in Image 1a. An increase in the amplitude of the whale calls recorded at the station results in a more accurate silhouette of the fin whale. At the peak amplitude of the seismic waveforms, the particles became more densely packed, allowing us to clearly see the characteristic shape of the fin whale portrayed by our artist colleagues. Utilizing spectrum analysis and dynamic imaging techniques on the seismic waveforms allows for the conversion of scientific information into artistic images and videos (Video S1).

Video S1. Art video created based on the seismic whale song recorded at the OBS station BS080

We further produced digital art videos totaling 10 min in length, based on the detection and extraction of 11 types of sounds in the Pacific Ocean (Figure S1), including whale songs, earthquake swarms, submarine eruptions, shipping, etc. The soundtracks and videos were carefully designed to capture the essence of the ocean soundscape while also delivering a visually pleasing and emotionally engaging experience.

#### Exhibition and public engagement

Our digital artworks were exhibited in several venues in 2023, attracting a diverse audience that included schoolchildren, scholars, artists, politicians, and the general public. We chose diverse methods to present our artworks to cater to different target audiences. At the Jinan International Biennale in early 2023, we showcased our videos and soundtracks utilizing high-resolution projectors and a professional audio array. This setup ensured that the sounds and visuals were perfectly synchronized, creating a harmonious experience for the audience. This was widely appreciated by both artists and the wider community. Our artworks attracted over 300,000 visits throughout the duration (3 months) of the Jinan International Biennale.

In June 2023, we participated in the science communication events on Ocean Science Day held by the Second Institute of Oceanography, Ministry of Natural Resources in China. We used a small monitor array to play our art videos. This option is not only cost-effective compared to professional projectors or video walls but also very convenient because it allows for easy switching between different sounds in the ocean on separate screens.

In addition, our digital artworks have been displayed alongside traditional art forms such as sculpture and light art. In November 2023, we curated a public exhibition on the interactions between earth science and art during the Blue Planet Science Fiction Film Festival in Nanjing, China. We used video wall systems to showcase our digital art videos depicting sounds in the ocean in a 450 square meter exhibition hall. In addition to our digital art videos, we also crafted sculptures symbolizing glaciers and built light installations representing devastating earthquakes and the rising sea level. This exhibition showcases a diverse range of artistic representations that demonstrate the potential of integrating science and art, as well as the underlying social values they embody. Furthermore, video documentary footage of the artworks has been incorporated into a dedicated webpage on a Chinese social media platform (in Chinese, www.zhihu.com/xen/market/ecom-page/1753104503861940225; Video S2), allowing users to access and watch the artworks online.

Video S2. Video documentary footage of our science-art exhibition in the Blue Planet Science Fiction Film Festival 2023, Nanjing, China

### Challenges and opportunities

#### Interpreting the world through art and science

While art and science are commonly perceived as distinct disciplines, they have a common objective of observing and interpreting the world. We expanded the ways in which data can be represented by combining science and art. This collaboration has resulted in the creation of digital artworks that not only capture the essence of the ocean soundscape but also deliver a visually pleasing and emotionally engaging experience. Our artworks highlight the potential of integrating artistic methodologies with scientific data to reveal the hidden realm of sounds in the ocean.

Moreover, the collaboration between the two fields has the potential to generate novel approaches, offering fresh perspectives and insights that foster further investigation.^12^^,^^13^ Our artworks provide new dimensions of scientific data representation, while the creation of the artworks is also driven by the analysis of scientific datasets. Due to technical constraints, our digital artworks were produced based on a limited number of sound detections in the ocean. Therefore, one of our ongoing tasks is to improve the capability of detecting and visualizing the sounds and/or events from extensive datasets using advanced techniques like artificial intelligence.

Moving forward, our goal is to broaden our collaborative endeavors to encompass additional domains within the field of geoscience. We hope to further integrate earth science and digital art in order to generate additional avenues for public engagement and education, ultimately cultivating a stronger bond between human societies and the ever-changing systems of our planet.

**Figure:**
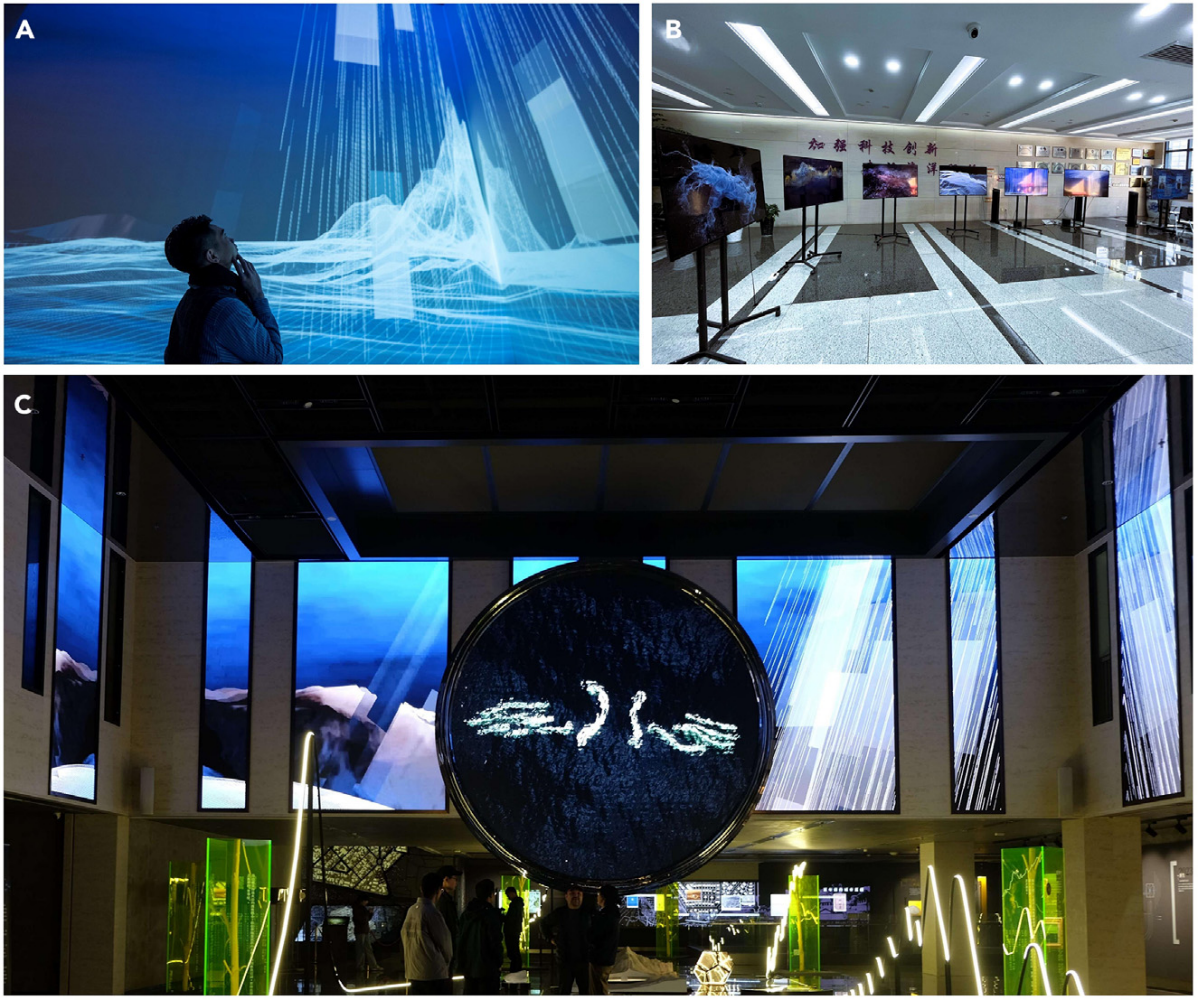
Digital artworks exhibited for various target audiences. Our team utilized (A) high-resolution projectors at the Jinan International Biennale, (B) a small monitor array for schoolchildren on Ocean Science Day 2023, and (C) video wall systems at a science-art exhibition in the Blue Planet Science Fiction Film Festival 2023, to showcase our digital art videos corresponding to different sources of sound in the ocean.

## Acknowledgments

To learn more about our recent artworks, please visit www.instagram.com/deep_see_light/. Our science-art project benefited from the 2022 Beacon Prize sponsored by Zhihu Incorporation. Y.R. was supported by the China Scholarship Council (grant 201904910466). This manuscript benefited greatly from conversations with Helene-Sophie Hilbert, Sibiao Liu, and Maochuan Zhang.

## References

[1] Rodrigues J., Castro C., Costa e Silva E., Pereira D.I. (2023). Geoscientists' views about science communication: predicting willingness to communicate geoscience. Geosci. Commun..

[2] Crameri F., Shephard G.E., Heron P.J. (2020). The misuse of colour in science communication. Nat. Commun..

[3] Barrett N., Mair K. (2014). Aftershock: A science–art collaboration through sonification. Org. Sound.

[4] Wu T., Ren Y., Liu S., Li C., Yao P., Hu X. (2023).

[5] Lu Z., Zhu X., Du X., Li J. (2024). Development and Prospect of Deep-Sea Environmental Noise Monitoring Technology. Earth Sci..

[6] Tyack P.L. (2008). Implications for marine mammals of large-scale changes in the marine acoustic environment. J. Mammal..

[7] Duarte C.M., Chapuis L., Collin S.P., Costa D.P., Devassy R.P., Eguiluz V.M., Erbe C., Gordon T.A.C., Halpern B.S., Harding H.R. (2021). The soundscape of the Anthropocene ocean. Science.

[8] Ren Y., Lange D., Grevemeyer I. (2023). Seismotectonics of the Blanco transform fault system, northeast Pacific: Evidence for an immature plate boundary. JGR. Solid Earth.

[9] Kuna V.M., Nábělek J.L. (2021). Seismic crustal imaging using fin whale songs. Science.

[10] Wilcock W.S.D., Hilmo R.S. (2021). A method for tracking blue whales (Balaenoptera musculus) with a widely spaced network of ocean bottom seismometers. PLoS One.

[11] Kilb D., Peng Z., Simpson D., Michael A., Fisher M., Rohrlick D. (2012). Listen, Watch, Learn: SeisSound Video Products. Seismol Res. Lett..

[12] Jacobsen E. (2016). Seismic wave videos combine sight and sound. Eos.

[13] Rees K.V. (2024). The soil zones of Saskatchewan: Creating art to visualize the concept. iScience.

