# Supplementary material for: Bridging science and art: Auralization and visualization of the ocean soundscape
Source: iScience. 2024 Aug 30;27(9):110696. doi: 10.1016/j.isci.2024.110696 (PMC11402209; doi:10.1016/j.isci.2024.110696)
Supplement: Document S1. Figure S1 [file mmc1.pdf]

iScience, Volume *27*

Supplemental information

Bridging science and art: Auralization and visualization of the ocean soundscape

Yu Ren, Tian Wu, Chuanzhi Li, Pengfei Yao, and Weiwei Ding

Supplemental information

Bridging Science and Art: Auralization and Visualization of Ocean Soundscape

Authors: Yu Ren[1,4*], Tian Wu[2,4], Chuanzhi Li[3,4], Pengfei Yao[4], and Weiwei Ding[5,6]

Affiliations:

[1]GEOMAR Helmholtz Centre for Ocean Research Kiel, Kiel, Germany

[2]Muthesius University of Fine Arts and Design, Kiel, Germany

[3]Ocean University of China, Qingdao, China

[4]Deep Sea Light Team, Kiel, Germany

[5]Key Laboratory of Submarine Geosciences, Second Institute of Oceanography, Ministry of Natural Resources, Hangzhou, China

[6]School of Oceanography, Shanghai Jiao Tong University, Shanghai, China

*Corresponding author: Yu Ren (yren@geomar.de)

Supplementary Audio 1: Fin whale vocalizations recorded at OBS station BS080 in the northeast Pacific Ocean. Fin whale calls recorded by seismic stations in the northeast Pacific sped up 10 times to be audible to humans.

Supplementary Video 1: Art video created based on the seismic whale song recorded at the OBS station BS080. Video resolution is compressed to 480p due to file size limitations of the submission system.

Supplementary Video 2: Video documentary footage of our science-art exhibition in the Blue Planet Science Fiction Film Festival 2023, Nanjing, China. Video resolution is compressed to 480p due to file size limitations of the submission system.

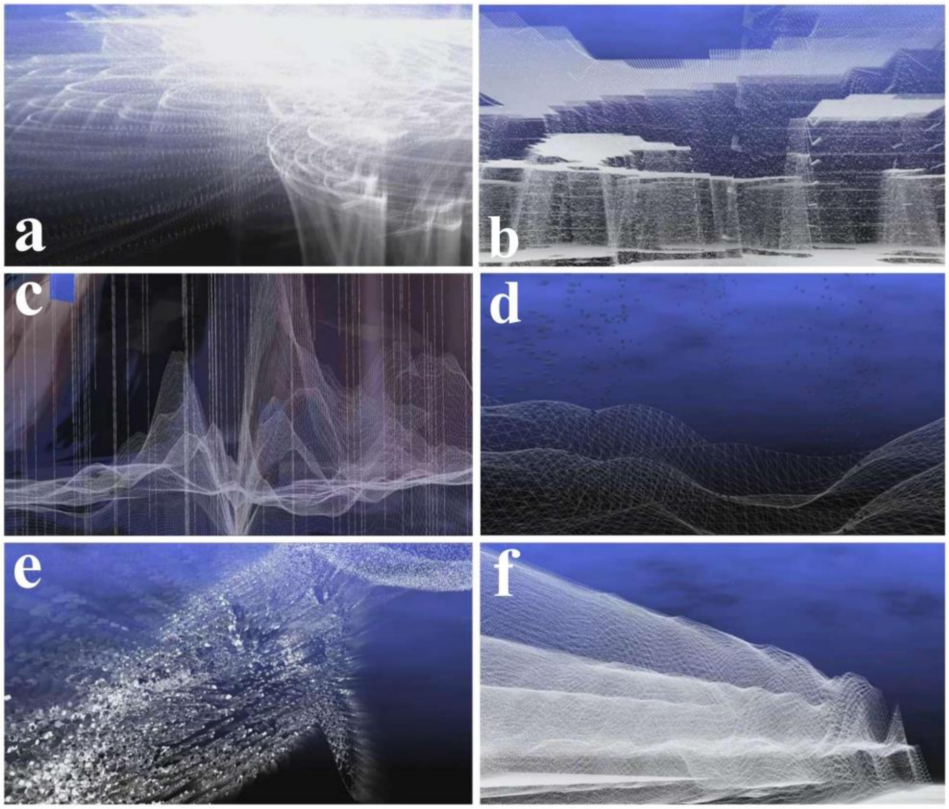

Supplementary Figure 1: Artistic representations of common sounds, such as **(a)** ship noise, **(b)** active seismic investigation, **(c)** earthquakes, **(d)** hydrothermal venting, **(e)** blue whale songs, and **(f)** large storms in the Pacific Ocean.
